# Hypertrophic olivary degeneration concomitant with bilateral middle cerebellar peduncles Wallerian degeneration following unilateral pontine infarction

**DOI:** 10.1186/s12883-020-01984-x

**Published:** 2020-11-07

**Authors:** Bing Bao, Xiangbin Wu, Zhongbin Xia, Yaoyao Shen

**Affiliations:** Department of Neurology, The Affiliated Hospital of Jiujiang University, No.57 Xunyang East Rode, Xunyang District, Jiujiang, 332000 Jiangxi Province China

**Keywords:** Wallerian degeneration, Middle cerebellar peduncles, Hypertrophic olivary degeneration, MRI

## Abstract

**Background:**

Wallerian degeneration (WD) can occur in different projecting systems, such as corticospinal tract, dentate-rubro-olivary pathway, and corticopontocerebellar tract. However, the co-occurrence of hypertrophic olivary degeneration (HOD) and middle cerebellar peduncles (MCPs) degeneration secondary to unilateral pontine infarction in a single patient is extremely rare.

**Case presentation:**

A 71-year-old man presented with acute onset of dizzness, slurred speech, and right-sided weakness. On the next day, his previous neurologic deficits deteriorated. Brain magnetic resonance imaging (MRI) revealed acute ischemic stroke of the left pons. After treatment with thrombolysis, antiplatelets, and rehabilitation training, his speaking and motor function improved moderately. At the 3-month follow-up, the MRI showed hyperintensity in the left medulla oblongata and bilateral MCPs on T2-weighted and FLAIR images, suggesting HOD as well as MCPs degeneration.

**Conclusions:**

It is of great importance for us to know the anatomic knowledge of dentate-rubro-olivary and corticopontocerebellar pathways.

## Background

Wallerian degeneration (WD) refers to the progressive anterograde disintegration of axons and accompanying demyelination following injury to the axon or cell body. It can be caused by a wide spectrum of diseases, such as cerebrovascular diseases, neoplasms, hemorrhage, surgery, epilepsy, and white matter diseases [1]. As its large number of axons and vital functions, the corticospinal tract has been widely recognized and extensively studied in relation to WD. Apart from corticospinal tract, WD can occur in other projecting systems, including corticopontocerebellar tract, dentate-rubro-olivary pathway, posterior column of the spinal cord, corpus callosum, limbic circuit, and optic pathway [2]. Previously, WD of the middle cerebellar peduncles (MCPs) following pontine infarction has been rarely depicted on conventional magnetic resonance imaging (MRI) studies. The MCP is vulnerable to WD because it is the largest and the the main path for pontocerebellar tracts. Bilateral and symmetrical hyperintensities along the MCPs on T2-weighted and fluid-attenuated inversion recovery (FLAIR) images are obviously detectable 2–3 months after stroke [3]. The dentate-rubro-olivary pathway, also known as Triangle of Guillain-Mollaret, is an important neural circuit involved in modulation of spinal cord motor activity. Hypertrophic olivary degeneration (HOD), another classic example of WD, characterized by trans-synaptic degeneration caused by a lesion involving in the dentate-rubro-olivary pathway. On MRI, HOD manifests as an increased T2/FLAIR signal intensity and enlargement of the inferior olive. HOD has various etiologies, including posterior fossa surgery, tumor, hemorrhage, infarction, infectious or infammatory processes, and traumatic brain injury. To the best of our knowledge, the co-occurrence of hypertrophic olivary degeneration (HOD) and the middle cerebellar peduncles (MCPs) WD secondary to unilateral pontine infarction reported in a single patient is extremely rare [4].

## Case presentation

**Fig. 1:**
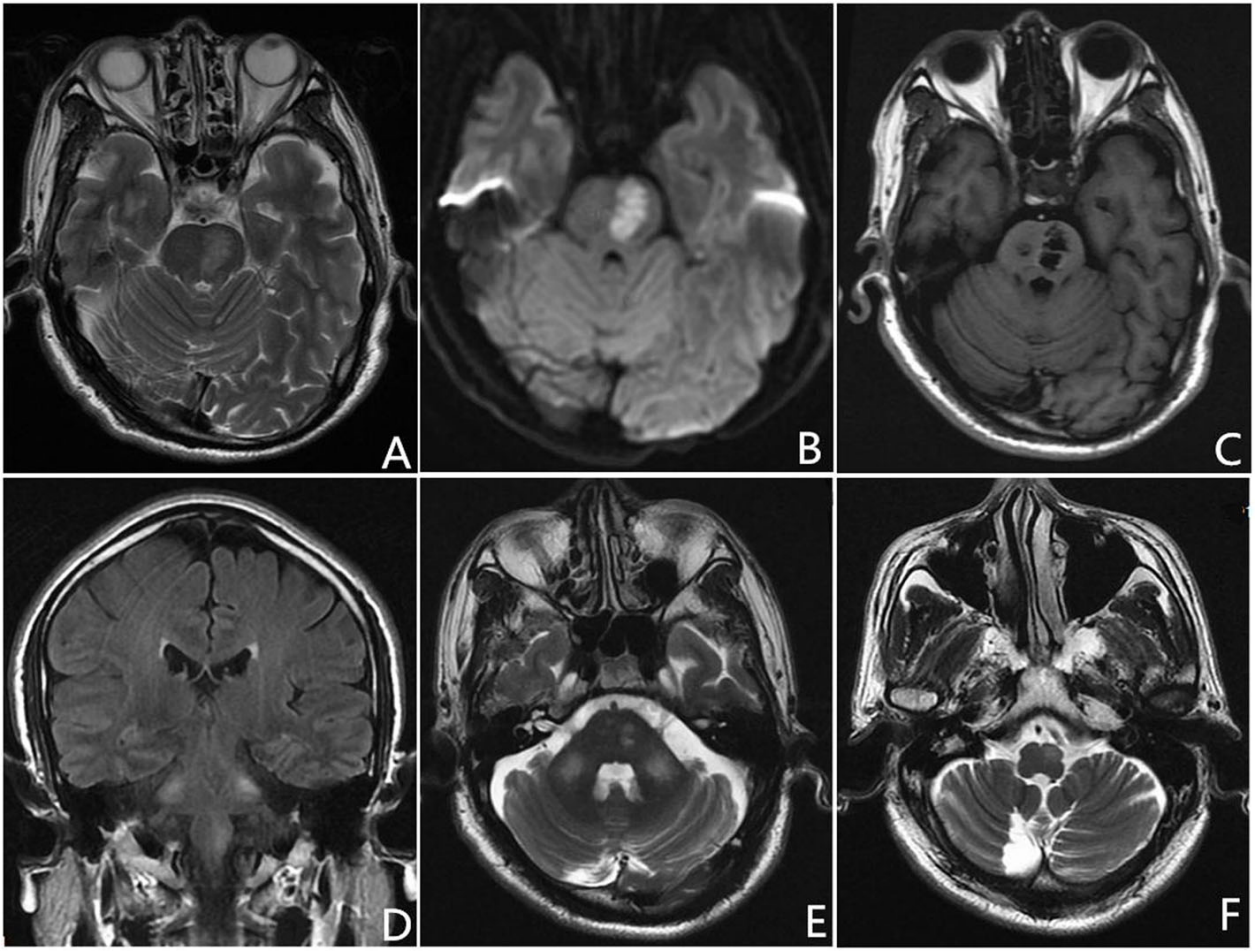
Axial T2-weighted and DWI images show acute pontine infarction (**a**, **b**). On 3-month follow-up MRI, axial T1-weighted image demonstrates an old paramedine pontine infarction (**c**). Coronal fluid-attenuated inversion recovery and axial T2-weighted images reveal hyperintensiy in the left inferior olivary nucleus (ION) and bilateral middle cerebellar peduncles (MCPs) (**d**-**f**)

A 71-year-old man (weight 60 kg) with known long-standing hypertension and smoking presented with acute onset of dizzness, slurred speech, and right-sided weakness for 1.5 h. Upon admission, the blood pressure was 170/100 mmHg, and the heart rate was 80 beats/min and regular. Neurological examination revealed dysarthria, left facial paralysis, hemiparesis and thermal/pain hypesthesia in his right limbs. The score on the National Institutes of Health Stroke Scale (NIHSS) was 5. An noncontrast computed tomography (CT) of head showed normal findings. Thrombolysis was initiated with 54 mg intravenous tissue plasminogen activator (rt-PA) 2 h after onset. After rt-PA, his previous neurologic deficits deteriorated (NIHSS score, 11). Brain MRI disclosed acute ischemic stroke of the left pons (Fig. 1a, b). CT angiography showed about 60% stenosis of the middle segment of basilar artery. All serum laboratory investigations were normal. On day 2 after admission, our patient was administrated with antiplatelets, antihypertensives, and rehabilitative treatment. After speaking and motor function improved moderately, he was discharged with a modified Rankin Scale (mRS) score of 2 on day 20 after admission.

Three month later, the patient was admitted to our department again for follow-up. No tremor, including palatal tremor, was observed. A repeated MRI showed previous pontine infarction (Fig. 1c). Besides, increased signal intensity was noted in the left medulla oblongata and bilateral MCPs on T2-weighted and FLAIR images (Fig. 1d-f), consistent with HOD and bilateral MCPs degeneration.

## Discussion and conclusions

**Fig. 2:**
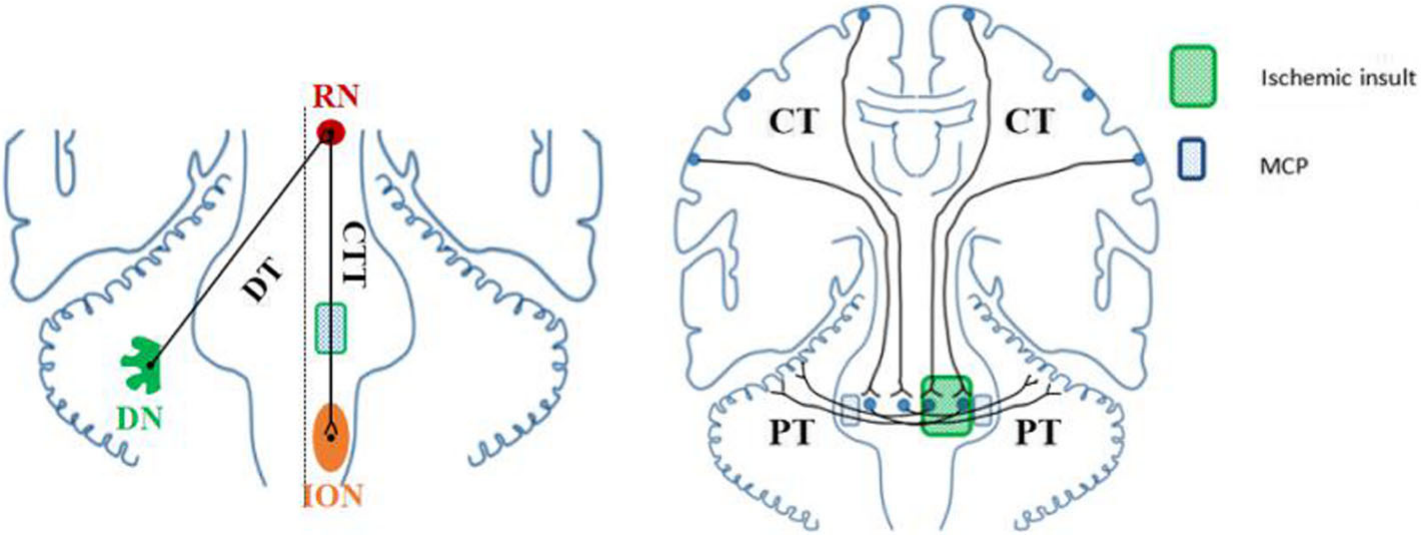
Schematic drawing illustrating of hypertrophic olivary degeneration (left) and middle cerebellar peduncles degeneration (right) (the image is depicted by ourselves). Abbreviations: DN, dentate nucleus; DT, dentatorubral tract; RN, red nucleus; CTT, central tegmental tract; ION, inferior olivary nucleus; CT, corticopontocerebellar tract; PT, pontocerebellar tracts; MCP, middle cerebellar peduncle

To our knowledge, hyperintensity on T2-weighted imaging with mild hypertrophy of the inferior olivary nucleus (ION) in the medulla oblongata is consistent with hypertrophic olivary degeneration (HOD), which usually occurs following a lesion involving the dentato-rubro-olivary pathway within the triangle of Guillain and Mollaret (GMT) [5]. Anatomically, afferent fibers originated in the dentate nucleus of the cerebellum ascend through the dentatorubral tract in the superior cerebellar peduncle and reach the contralateral red nucleus (RN) in midbrain across midline. Afterwards, the efferent fibers from the RN descend through the central tegmental tract to enter into the ipsilateral ION (Fig. 2). Any components involvement interrupting the pathway from dentate nucleus to ION may cause HOD. A wide spectrum of etiologies have been described, including cerebrovascular diseases, tumor, toxic, prior surgery, encephalitis, neuro-Behçet’s disease, or Wilson disease [6]. Among those, cavernous malformation, ischemic infarction, and hemorrhage are the most common causes. In our patient, pontine infarction interrupts the efferent fibers from the RN to enter into ipsilateral ION, resulting in ipsilateral HOD. The classical clinical presentation of HOD is palatal tremor, which is characterised by rhythmic involuntary movements of the oropharynx due to contractions of the levator veli palatini muscle. The frequency of palatal tremor is estimated to be 33.8% in HOD patients [5]. Hence, palatal tremor can be absent in HOD.

It is worth noting that hyperintensity in bilateral MCP on T2-weighted imaging can also be noted in this case. The MCPs are mainly composed of pontocerebellar tracts that connect the basal portion of the pons with the cerebellum. By routine MRI techniques, normal MCPs demonstrate homogeneous white matter signal intensity. Previously, classification of diseases that manifest as symmetrical lesions on the MCPs includes neurodegenerative diseases, toxic or metabolic diseases, cerebrovascular diseases and demyelinating diseases [3]. Bilateral MCPs WD secondary to pontine infarction has been described [7]. Pontocerebellar tracts arise from the controlateral pontine nuclei which receive cortico-pontine tracts. They cross the midline at an upper pontine level and pass through the MCP to reach the cerebellar cortex (Fig. 2). When a lesion occurs in one side of the pons, as we report here, homolateral pontine nuclei and the contralateral pontocerebellar tracts simultaneously involved, resulting in bilateral MCPs WD.

MRI sequences have been used to depict changes of WD in different stages, especially for corticospinal tract. Histologic and metabolic features on different stages of WD are in accordance with specific findings on MRI [8]. The first stage is described by disintegration of the axons and slightly biochemical change in myelin within 4 weeks after injury. No signal intensity abnormalities are usually recognizable. The second stage is characterized by the rapid destruction of the myelin sheath from 4 to 14 weeks after stroke. As the tissue becomes more hydrophobic accompanying by myelin-protein breakdown, the high lipid-protein ratio leading to hypointense on T2-weighted image. In the third stage, with subsequent myelin lipid breakdown, gliosis and changes in water content and structure, resulting in increased hydrophilicity and hyperintensity on T2-weighted and FLAIR images. The fourth stage is characterized by volume loss and atrophy in the brainstem, which occur few years later. Therefore, the best time to undergo follow-up MRI examination may be at least 4 weeks when a patient is diagnosed with pontine infarction.

In conclusion, HOD concomitant with bilateral MCPs WD in the setting of unilateral pontine infarction is extremely rare. It is of great importance for us to know the anatomic knowledge of dentate-rubro-olivary and corticopontocerebellar pathways.

## Data Availability

Not applicable.

## Abbreviations

MRI: Magnetic resonance imaging
WD: Wallerian degeneration
HOD: Hypertrophic olivary degeneration
MCPs: Middle cerebellar peduncles
NIHSS: National Institutes of Health Stroke Scale
CT: Computed tomography
rtPA: Recombinant tissue plasminogen activator
mRS: Modified Rankin Scale
FLAIR: Fluid-attenuated inversion recovery
ION: Inferior olivary nucleus
GMT: Guillain and Mollaret
RN: Red nucleus
